# Supplementary material for: SARS-CoV-2 Serosurveillance Reveals Pre-pandemic Cross-Reactivity and Pandemic Seroprevalence Trends in Senegal
Source: medRxiv. 2025 Oct 21:2025.10.20.25337295. Preprint. [Version 1] doi: 10.1101/2025.10.20.25337295 (PMC12633569; doi:10.1101/2025.10.20.25337295)
Supplement: Supplement 1 [file media-1.docx]

**SARS-CoV-2 Serosurveillance Reveals Pre-pandemic Cross-Reactivity and Pandemic Seroprevalence Trends in Senegal**

Mouhamad Sy^1,2^, Ian Baudi^3^, Ibrahima M. Ndiaye^1^, Mariama Toure^1^, Amy Gaye^1^, Tolla Ndiaye^1^, Aida S. Badiane^1,4^, Awa B. Deme^1^, Jules Gomis^1^, Daba Zoumarou^1^, Mouhamadou Ndiaye^1,4^, Khadim Diongue^1,4^, Mame Cheikh Seck^1,4^, Djiby Sow^1,4^, Ngayo Sy^5^, Mouhamadou A. Diallo^1,4^, Marietou F. Paye^3^, Pardis C. Sabeti^3^, Katherine J. Siddle^2,3^, Daouda Ndiaye^1,4^

1 International Research and Training Center for Applied Genomics and Health Surveillance (CIGASS) at UCAD, Dakar, Senegal.

2 Department of Molecular Microbiology and Immunology, Brown University, Providence, RI, USA

3 Broad Institute of MIT and Harvard, Cambridge, MA, United States

4 Department of Parasitology, Faculty of Medicine, University Cheikh Anta Diop, Dakar, Senegal

5 Service de Lutte Antiparasitaire (SLAP) de Thies, MoH, Senegal

## **Supplementary or exploratory figure/table alternatives**


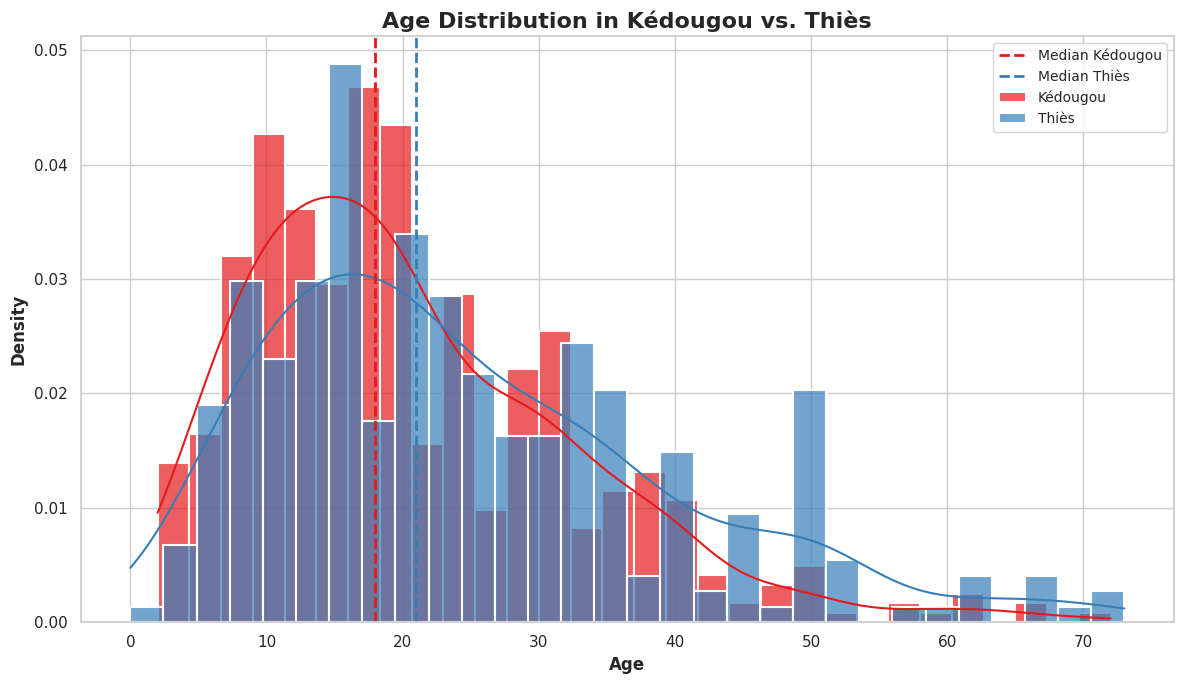


Sup. Fig 1: Overall age distribution
